# The complete mitochondrial genome of *Entylia carinata* (Hemiptera: Membracidae)

**DOI:** 10.1080/23802359.2016.1219629

**Published:** 2016-09-04

**Authors:** Meng Mao, Xiushuai Yang, Gordon Bennett

**Affiliations:** Department of Plant and Environmental Protection Sciences, University of Hawaii, Manoa, Honolulu, HI, USA

**Keywords:** *Entylia carinata;* Membracidae, mitochondrial genome, tandem repeat

## Abstract

The complete mitochondrial genome of the treehopper species, *Entylia carinata,* was sequenced using the next-generation sequencing method. The completely assembled mitochondrial genome is 15,483 bp long. The genome follows the typical invertebrate mitochondrial gene arrangement, and includes 13 protein-coding genes, 22 tRNA genes, two rRNA genes and one A + T-rich region. Two tandem repeats were also identified within the A + T-rich region.

The treehopper family, Membracidae, comprises 400 genera and ∼3500 described species distributed globally (Cryan et al. 2004; Deitz & Wallace 2010, 2012). Membracidae exhibits several remarkable life-history traits, including brood care and ant-tending mutualisms (Deitz & Wallace 2010). However, species-level phylogenies and resources for molecular systematics remain limited for this group. The lack of molecular markers hinders research questions that address species phylogeny, the evolution of life-history traits and ecological adaptations, and historical biogeography. To provide additional membracid resources, we sequenced the complete mt genome of *Entylia carinata*. This species has a widespread distribution across the North American continent (McKamey 1998; Godoy et al. 2006; Deitz & Wallace 2010). *Entylia carinata* is a polyphagous phloem-sap feeder that relies on microbial symbionts for nutritional supplementation of its herbaceous plant diet (Buchner 1965; Wood 1993).

Insect specimens were collected in 2013 from Yale Campus, Orange, Connecticut, U.S.A (41°15′16.3″N, 72°59′33.4″W) and deposited in the University of Hawaii Insect Museum (Accession Number: UHIM2016-4430, UHIM2016-4431). Purified genomic DNA was extracted with a Qiagen DNeasy kit and sequenced at the University of Texas, Austin GSAF. Genomic libraries were prepared from 300 base pair (bp) fragments and sequenced on an Illumina MiSeq (2 × 300 bp PE reads). A single full-length contig was obtained for the mt genome with SPAdes V3.6.2 (Bankevich et al. 2012). Completeness was assessed by read mapping with Bowtie2 (Langmead & Salzberg 2012) to confirm that no coverage breaks exist. Protein-coding genes (PCGs), rRNAs, and tRNAs were annotated in Geneious v9.0.2 (Kearse et al. 2012). Gene predictions were verified with related mt genomes (Figure 1). The complete mt genome of *E. carinata* is 15,483 bp (GenBank no. KX495488) with 168× the average read coverage. We identified 37 genes (13 PCGs, 22 tRNAs, and 2 rRNAs) and the A + T-rich region.

**Figure 1.**
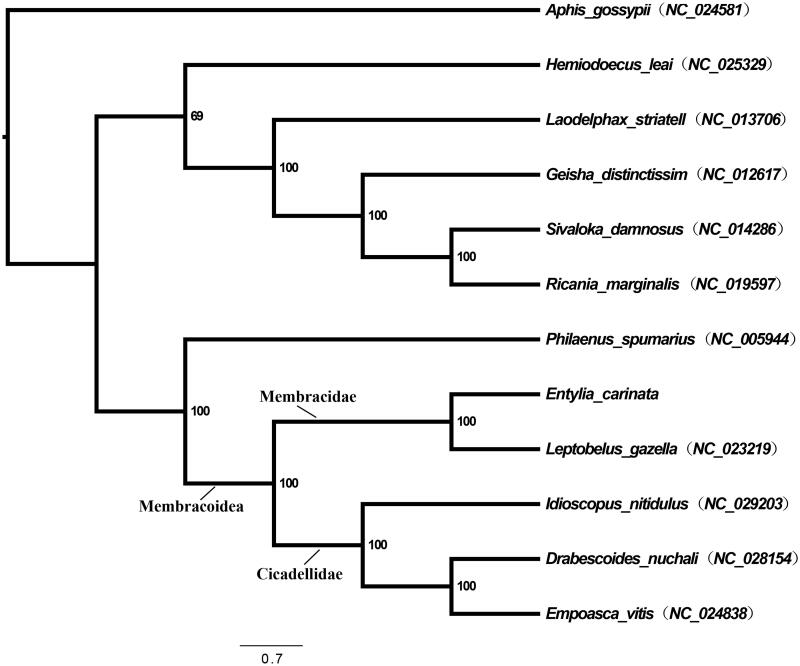
Maximum-likelihood phylogeny of Hemiptera species with fully sequenced mitochondrial genomes. Phylogenetic reconstructions were done from a concatenated matrix of all 13 protein-coding mitochondrial genes with RAxML-HPC2 under the GTRCAT model in the CIPRES portal (Miller et al. 2010; Stamatakis 2014).

The nucleotide composition of *E. carinata* has a biased A + T content of 78.1% for the majority strand (J-strand), which is much lower than the related *Leptobelus gazella* (79.4%; Membracidae) (Zhao & Liang 2016). All 13 PCGs start with ATN codons. Nine PCGs (*ATP6*, *ATP8*, *CYTB*, *ND1*, *ND2*, *ND3*, *ND4*, *ND4L*, and *ND6*) use the typical stop-codons (TAA or TAG), while the remaining four PCGs (*COX1*, *COX2*, *COX3*, and *ND5*) use the incomplete T stop-codon. The putative A + T-rich region is 1474 bp long (79.4% A + T content). Finally, two tandem repeats were observed in the A + T-rich region, which were shared with *L. gazella*.

To verify the phylogenetic placement of *E. carinata* within the Auchenorrhyncha (Hemiptera), a maximum-likelihood phylogeny with 13 PCGs was reconstructed in RAxML-HPC2 (GTRCAT model) in CIPRES (Miller et al. 2010; Stamatakis 2014). Phylogenetic relationships validate the position of *E. carinata* within the Auchenorrhyncha and also within Membracidae (Figure 1). Furthermore, both *E. carinata* and *L. gazella* are placed in a monophyletic group with leafhoppers (Cicadellidae). This result supports previous work indicating the close – possibly paraphyletic – relationship between treehoppers and leafhoppers within Membracoidea (Cryan et al. 2004; Cryan & Urban 2011).
